# Bioaccumulation and physiological changes in the fruiting body of *Agaricus bisporus* (Large) sing in response to cadmium

**DOI:** 10.1038/s41598-022-24561-3

**Published:** 2022-11-22

**Authors:** Haiyan He, Dan Zhang, Jianing Gao

**Affiliations:** 1 College of Geography and Resources Science, Neijiang Normal University, Neijiang, People’s Republic of China; 2 Key Laboratory of Mountain Surface Processes and Ecological Regulation, Institute of Mountain Hazards and Environment, Chinese Academy of Sciences, Chengdu, People’s Republic of China

**Keywords:** Environmental sciences, Metals

## Abstract

The bioremediation of heavy metals contaminated soils with macrofungi is a new and promising approach; hence *Agaricus bisporus* (Large) sing has potentially shown accumulating ability to Cd contamination. This study focused on the tolerance response by *A. bisporus* to different contents of Cd in the closed cup and the flat stage of fruiting body development. The contents of Cd, soluble protein, sugar, low molecular weight organic acids (LMWOAs), and antioxidant activity were investigated. The bioaccumulation factor and transfer factor results revealed that Cd accumulated in the cap of *A. bisporus* more than that in the stipe with the highest content being 18.38 mg kg^−1^ dry weight at the closed cup stage under 414.28 mg kg^−1^ Cd stress. High Cd content stress increased soluble protein, proline, and malonaldehyde contents at both stages; while higher peroxidase, catalase, ascorbic acid peroxidase activities, and LMWOAs contents were only recorded at the closed cup stage. On the other hand, Superoxide dismutase activities and soluble sugar content showed a complex trend. Overall, these results have successfully established that *A. bisporus* could resort to modulating its metabolism to avoid the destructive effects of Cd stress and could successfully accumulate Cd in the soil, which is a promising prospect for the remediation of Cd-contaminated soils.

## Introduction

Cadmium (Cd) is one of the most toxic trace metallic elements for living organisms and its accumulation in the environment has long been recognized as a worldwide concern^1^. Over the past five decades, the worldwide release of Cd reached 22,000 t^2^. According to the investigation bulletin of soil pollution in China published in 2014, the over-standard rate of Cd is the highest and has reached 7.0%, hence is determined as the primary soil pollutant in China. Consequently, remediation of the heavy metal in contaminated soils has become urgent. Its efficient remediation technology has been the research hotspot with many difficulties. Remediation of heavy metals in contaminated soils is done by physical and chemical processes which are costly, time-consuming, unsustainable^3^, and at risk of secondary pollution. Contingently, bioremediation has been considered to be a promising method. At present, the research mainly focuses on phytoremediation, such as *Thlaspi caerulescens*^4^, *Sedum plumbizincicola*^5^, *Pteris vittata* L.^6^, *Solanum nigrum* L.^7^, which can be super-enriched heavy metals such as Cd, lead (Pb), zinc (Zn) and so on from soil. Phytoremediation depends on biological process, which requires a long period of remediation, much biomass^8^, and has seasonal limitations; thus, restricting large-scale application. The enrichment of heavy metals in macrofungi was first discovered from the high level of Cd accumulation in *Agaricus*^9^. Many macrofungi can effectively absorb and accumulate heavy metals in their fruiting bodies^10–13^. Mushrooms’ fruiting bodies are considered to be advantageous to plants as they have a shorter life cycle and better adjustment ability than plants^14^, Hence bioremediation via macrofungi is a novel and promising approach for heavy metals remediation^15^.

However, there are many problems in the remediation of heavily metals contaminated soils by macrofungi. For example, many wild macrofungi are not acclimated and are difficult to cultivate, with limited heavy metals tolerance and hyperaccumulating abilities as well^16^. Thus, the key factor for fungal repair application is to obtain a fungus with strong tolerance to heavy metals that is easy to cultivate. *Agaricus bisporus* (Large) Sing is one of the most widely cultivated edible fungi in the world. It has a certain enrichment capacity for cadmium^17^ and can be used as a cost-effective, efficient biosorbent for the removal of Cd (II) and Pb (II) from aqueous synthetic solutions^18^. It has the potential to be used as a material to repair Cd pollution. However, the lack of information on cultivated *A. bisporus* in Cd-contaminated soil made an impetus to undertake the present investigation. The present study was undertaken to investigate the bioremediation potential of *A.bisporus* with Cd and the effect of Cd on the physiological and biochemical functions of fruiting bodies with a view to the possible use of this mushroom for bioremediation of Cd-contaminated soil.

## Materials and methods

### Fungus growth and harvest under Cd stress

The fungus *A. bisporus* A15 used in this work was supplied by the Sichuan Academy of Agricultural Sciences, China. The cultivation experiment was carried out by frame culture with a 45 × 50 × 32 cm hollow plastic square frame. The casing soil was peat soil, with organic matter content of 455.65 ± 3.63 g kg^−1^, the total phosphorus content of 1.32 ± 0.14 g kg^−1^, the total nitrogen content of 15.69 ± 0.18 g kg^−1^, the total potassium content of 17.56 ± 0.66 g kg^−1^, the available potassium content of 924 ± 0.03 mg kg^−1^, the available phosphorus content of, 110.83 ± 16.03 mg kg^−1^ and available nitrogen content of 255.27 ± 7.92 mg kg^−1^. Before the experiment, the casing soil was air-dried and passed through a 5 mm diameter sieve to thoroughly remove stones and crop residues, artificially spiked with cadmium nitrate tetrahydrate (Cd(NO_3_)_2_·4H_2_O) as Cd (0, 20, 100, 500 mg kg^−1^, The corresponding numbers are CK, T1, T2, and T3). The Cd-treated casing soil will be placed at a moisture content of about 60% (added water and mixed every 10 days) for 50 days to make the distribution of Cd uniform and sampled to determine the actual Cd content before usage. The mushroom-growing experiment was conducted in the industrial production base of *A. bisporus*. The specific process is as follows: the *A. bisporus strain* (using kernel as cultural materials of the secondary fungus, which is prepared by the factory) was inoculated on the compost (about 15 cm high). The compost was mainly made of wheat straw, chicken manure, and rapeseed meal, with urea, gypsum, and peat soil added and fermented in two stages. The temperature and watering shall be controlled according to the actual situation to maintain the compost temperature at about 25 °C and the humidity at 90–95%. After 15 days, evenly cover treated peat soil of the different Cd contents with a thickness of about 3 cm and keep the compost temperature at about 25 °C and the humidity at 90–95%. When the mycelium grows to the soil surface, it starts to reduce the compost temperature from 25 °C to 20–21 °C and the relative humidity of air from 90–95% to 85–90% to promote fruit body growth. Sampling begins 23 days after soil covering. there are three replicates for each treatment (three plastic square frame).

### Sampling

The collected samples of *A. bisporus* were the first flush of mushrooms. The fruiting body was sampled at stages 3 (Closed cup stage) and 7 (Flat stage) of fruiting body development^19^ and each treatment was taken a mixed sample from each plastic frame. After sampling, the fruiting bodies were washed with ultrapure water and the caps and stipes were separated. The collected fungus samples were divided into three parts, some of which were dried to constant weight at 60 °C, crushed through a 60-mesh sieve, and stored in a dryer; and the others were weighed and stored directly at − 20 °C and − 80 °C respectively for further experiments.

### Detection of Cd content in soil and *A. bisporus* fruiting body

The soil and the *A. bisporus* fruiting body were respectively digested with concentrated sulfuric acid and nitric acid, each followed by a hydrogen peroxide solution. The quantitative determination of Cd (mg kg^−1^ DW) was carried out by ICP-OES (ICAP6300, Thermo Elemental).

### Soluble sugar and protein content analysis

The caps of *A. bisporus* stored at − 20 °C were taken for determination of soluble sugar and protein content. The Bradford method was used for protein quantification^20^: About 0.5 g sample was homogenized with 5.00 mL ultrapure water and incubated at 25 °C for 30 min. After centrifugation (20 min, 6000 rpm), 0.2 mL supernatant was added to 0.8 mL ultrapure water for further analysis. Bovine serum albumin as a standard sample, absorbance was determined at 595 nm and expressed in mg g^−1^ fresh weight (FW). Soluble sugar content was determined by refractometry with anthrone reagent: about 0.5 g sample was homogenized with 5.00 mL ultrapure water, incubated at 100 °C for 30 min, and then cooled to ambient temperature. After centrifugation (20 min, 6000 rpm), 0.1 mL supernatant was added to 0.9 mL ultrapure water for further analysis. Sucrose as a standard sample, the contents were calculated by absorbance at 630 nm and expressed in mg g^−1^ FW**.**

### Superoxide dismutase (SOD), peroxidase (POD), catalase (CAT), ascorbic acid peroxidase (APX) activity analysis

The caps of *A. bisporus* samples stored at − 80 °C were taken for determination of enzyme activity. The enzyme was extracted by grinding 0.5 g samples in liquid nitrogen. Then, the resultant powder was suspended in 5 mL 0.2 M potassium phosphate buffer (pH 7.8). After centrifugation (4 °C, 20 min, 10,000×*g*), the supernatant was preserved at − 4 °C for further analysis. SOD activity was measured by the nitro-blue tetrazolium (NBT) reduction method at 560 nm^21,22^. CAT activity was determined by measuring the decrease of H_2_O_2_ at 240 nm^21,23^. APX activity was estimated following the H_2_O_2_-dependent oxidation of ascorbate as a decrease at 290 nm^21,24^. POD activity was determined by measuring the increase in absorbance at 470 nm as a result of Guaiacol oxidation^16,25^. The enzyme activity was calculated by FW.

### Free proline and malonaldehyde (MDA) content analysis

The caps of *A. bisporus* samples stored at − 20 °C were taken for determination of proline and MDA content. To determine the free proline content: about 0.5 g sample was homogenized with 4 mL 3% sulfosalicylic acid solution, incubated at 100 °C for 10 min, then cooled to ambient temperature and centrifuged (20 min, 6000 rpm). The supernatants were used for free proline analysis according to the procedure described below^26,27^. Briefly, the 0.2 mL supernatant was added to 1.8 mL 3% sulfosalicylic acid solution, placed in a plastic test tube (15 mL), treated with ninhydrin acid reagent (2 mL) and glacial acetic acid (2 mL), heated for 30 min in a water bathtub at 100 °C, quickly cooled in an ice-cold water bathtub and then added toluene (4 mL) to each sample. Test tubes were sealed, vortexed for 15 s, and left at room temperature until the upper toluene layer with proline was separated from the lower water layer. l-proline as a standard sample, the content was calculated by absorbance at 520 nm. The final results were expressed as μg g^−1^ FW.

To determine the MDA content: About 1.0 g sample was homogenized with 4 mL 5% trichloroacetic acid (TCA) solution and centrifuged (20 min, 6000 rpm). The supernatant (2 mL) was added to 0.67% thiobarbituric acid (TBA) and heated (100 °C, 15 min), quickly cooled in an ice-cold water bathtub, and then centrifuged (10 min, 6000 rpm). Absorbance was determined at 450 nm, 532 nm, and 600 nm, and MDA content was calculated via the difference between both absorbance values^28^.

### Low molecular weight organic acids (LMWOAs) content analysis

The stipes of *A. bisporus* samples stored at − 20 °C were taken for determination of LMWOAs content. About 0.5 g sample was homogenized with 5.00 mL 0.1% H_3_PO_4_ and ultrasonic for 30 min and incubated at 75 °C for 15 min. After centrifugation (20 min, 13,000 rpm), the supernatant was passed through a 0.22 μm filter membrane for further high-performance liquid chromatography (HPLC) analysis^29^.

Method: Venusil MP C18 column (250 mm × 4.6 mm, 5 mm) was used with the mobile phase being 10 mmol L^−1^ KH_2_PO_4_ (pH 2.5) and methanol (V: V = 98:2), the flow rate was 0.5 mL min^−1^, detecting wavelength was 210 nm and 30 °C of column temperature. The sampling volume was 10 μL. The standard samples of oxalic acid, tartaric acid, malic acid, acetic acid, citric acid, fumaric acid, and succinic acid are all analytical pure. External calibration of peak area versus content was used for quantification and expressed in mg g^−1^ FW.

### Statistical analysis

All detection indicators were evaluated by a double-factorial analysis of variance including the Cd stress content and sampling time. Statistical analysis was performed with SPSS 19.0 software. All data were the means ± SD of three independent replicates. Fisher’s protected least significant differences (LSD) at a significance level of P < 0.05 were used for multiple comparisons. All figures were performed using Microsoft Excel 2010.

The bioconcentration factors (BCF) were calculated by Eq. (1) and the transfer factors (TF) were calculated by Eq. (2)^30^.

1 $$\mathrm{BCF}=\frac{\mathrm{Cd \; content \; in \; the \; fruiting \;body}}{\mathrm{Cd \;content \;in \;the \;soil}}$$

2 $$\mathrm{TF}=\frac{\mathrm{Cd \;content \; in \; the \;cap}}{\mathrm{Cd \; content\; in \;the\; stipe}}$$

## Results and discussion

### Effect of Cd stress on the Cd bioaccumulation, BCF, and TF in the fruiting body of *A. bisporus*

The theoretical addition and measured content of Cd in the casing soil of *A. bisporus* cultivation are shown in Table 1.

**Table 1:** The theoretical addition and actual concentration of Cd in the casing soil (DW).

No	Theoretical addition (mg kg^−1^)	Measured value (mg kg^−1^)
CK	0.00	0.44 ± 0.04
T1	20.00	17.44 ± 0.39
T2	100.00	107.42 ± 2.07
T3	500.00	414.28 ± 0.12

**Figure 1:**
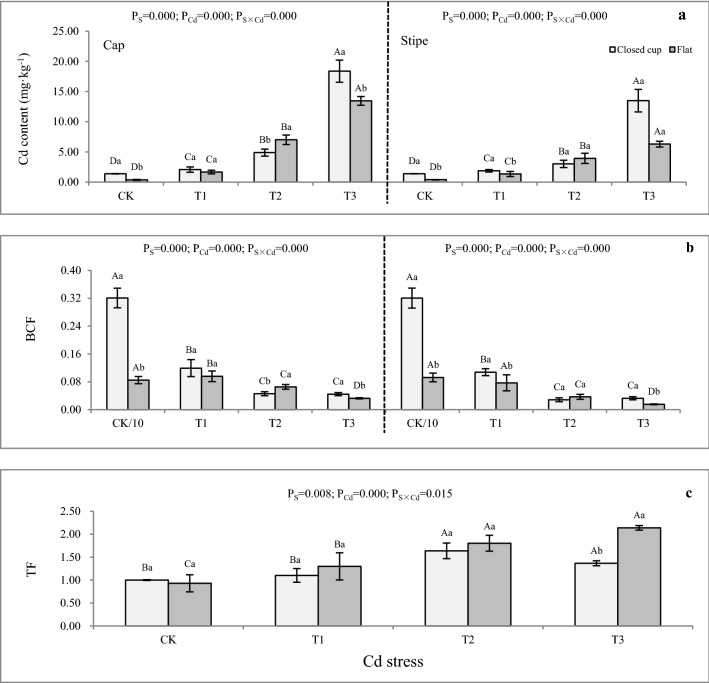
Cd concentration, BCF and TF in fruiting bodies of *A. bisporus*. (**a**) Cd concentration in the cap and stipe. (**b**) BCF in the cap and stipe. (**c**) TF in the fruiting body. Different capital letters indicate significant differences among Cd stresses under the same sampling stage determined by LSD’s test with p < 0.05; different lower letters indicate significant differences within the sampling stage under the same Cd stress concentration determined by LSD’s test with p < 0.05. P_S_: effect of sampling time; P_Cd_: effect of Cd concentration; P_S×Cd_: Interaction between sampling time and Cd concentration. Error bar: SD.

The Cd contents were 0.37–18.38 mg kg^−1^ DW in the cap and 0.40–13.50 mg kg^−1^ DW in the stipe, indicating *A. bisporus* could accumulate Cd. Similar to the previous studies: the BCF of *A. bisporus* cultivated on humic compost artificially fortified with Cd was higher than 1^31^. *A. bisporus* planted in plastic pots full of soil fortified with different concentrations of Cu^2+^, Zn^2+^ and Cd^2+^ were found to tolerate all the added heavy metals, but only bioconcentrate Cd (BCF > 1.0)^32^. The Cd bioaccumulation of *A. bisporus* showed a significant Cd stress content-dependent trend and gradually increased (Fig. 1a). The BCFs in the cap and stipe were 0.03–3.21 and 0.02–3.20 respectively, and the changing trend was opposite to that in Cd accumulation (Fig. 1b). In the T1–T3, the Cd content and BCF in the caps were higher than those in the stipe, which were also similar to previous reports^33^. The Cu bioaccumulation in the cap of *Oudemansiella radicata* was higher than that in the stipe and gradually increased with the rise of Cu stress content^33^. The highest Cd content was 18.38 mg kg^−1^ DW in the cap, recorded in the closed cup stage of T3, while the maximum BCF was 3.21 in the cap at the close upstage of CK (Fig. 1a,b). The BCF of CK was 0.85–3.21, which was higher than those of T1-T3, indicating that *A. bisporus* could effectively extract Cd from the soil at a low Cd pollution level. The TFs of *A. bisporus* was 0.93–2.14 (Fig. 1c), and greater than 1 except for CK, suggesting that Cd accumulation in *A. bisporus* shifted from bottom to top. The biomass of the *A. bisporus* cap is higher than that in the stipe, which is beneficial to the extraction of Cd from the soil. Except for CK, the *A. bisporus* had lower Cd accumulation than the soil substrates, thus resulting in a relatively low BCF. The reason may be due to the short growing-time of the fruiting body^34^. However, *A. bisporus* can survive in high Cd content stress and accumulate high Cd content in a short lifetime, indicating its toleration potentialities in a Cd-contaminated soil environment.

### Effect of Cd on the content of soluble protein and sugar

Heavy metal stress will inevitably lead to physiological and biochemical changes in the body. The change in protein content is one of the physiological responses of organisms to heavy metals stress. The protein content could be increased, unchanged, or decreased^35,36^, but is usually manifested as a promoter of protein synthesis at a low level and an inhibitor at a high level. For example, the 5–40 mg L^−1^ Cd promotes the protein synthesis of *Escherichia coli*, *Bacillus subtilis*, *Saccharomyces cerevisiae* Hansen and *Streptomycetaceae*, and then inhibits the protein synthesis at 50–100 mg L^−1^ Cd (80–100 mg L^−1^ in *Streptomycetaceae*)^37^.

**Figure 2:**
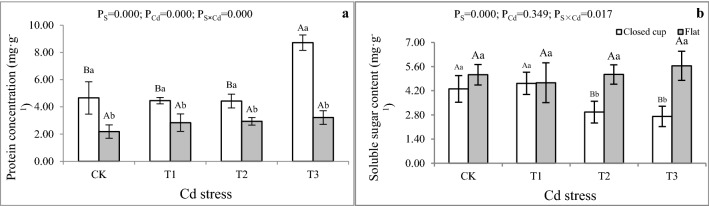
Effect of Cd stress on the content of soluble protein and sugar in the fruiting bodies of *A. bisporus*. (**a**) Soluble protein content. (**b**) Soluble sugar content. Different capital letters indicate significant differences among Cd stresses under the same sampling stage determined by LSD’s test with p < 0.05; different lower letters indicate significant differences within the sampling stage under the same Cd stress concentration determined by LSD’s test with p < 0.05. P_S_: effect of sampling time; P_Cd_: effect of Cd concentration; P_S×Cd_: Interaction between sampling time and Cd concentration. Error bar: SD.

In the study, as shown in Fig. 2a, the protein contents in the cap of *A bisporus* in the closed cup stage were significantly higher than that in the flat stage (p < 0.05) in the T1-T3. In the closed cup stage, the protein content decreased with the increase of Cd stress. The highest protein content was 8.72 mg g^−1^ FW in the T3, which was significantly higher than the other three groups and 87.24% higher than in the CK. However, the protein content inconsiderably increased with the increase of Cd stress content in the flat stage. Similar results are found in *Agaricus brasiliensis* strain J1^38^. The changes in protein content were independent of stress content without a significant change. The reason may be that *A. bisporus* synthesizes some specific proteins or polypeptides that bind to Cd to reduce the damage of Cd, similarly to plants^5^. In addition, *A. bisporus* may exhibit various regulatory properties at different growth stages at different Cd stress levels. Similar results were found in rice under Zn or Cr stress^35^.

Soluble sugar, as the main osmotic agent, has a stabilizing effect on the cell membrane and protoplasmic colloid under heavy metals stress^39^. Most of the previous reports have found the content of soluble sugar in the body increased under heavy metals stress^35,40^. For example, With the increase of Cd concentration, polysaccharides of *A. brasiliensis* strain J1 showed an upward trend with a significant difference compared with the control treatment, which indicated that Cd concentration might promote the accumulation of polysaccharides of J1^38^. In the present study, the soluble sugar content in the closed-cup stage gradually decreased with the increase of Cd stress content, and only the T1 was higher than that of the CK, while the flat stage showed the opposite trend (Fig. 2b). The soluble sugar contents of *A. bisporus* in the closed cup stage were lower than that in the flat stage with a significant difference in T2 and T3 (Fig. 2b), which was contrary to the change in the trend of protein. The soluble sugar content of T3 reached a maximum value of 5.65 mg g^−1^ FW in the flat stage, which was 106.96% higher than that in the closed cup stage. Cd could inhibit the synthesis of soluble sugar under short Cd stress, but the content of soluble sugar could increase with the increase of stress time. The protein content of *A bisporus* in the closed cup stage was higher than that in the flat stage, while the soluble sugar content had the opposite change. The reason might be that under heavy metal stress, the absorption of some essential ions is weakened, resulting in the accelerated decomposition of substances such as starch, protein, and nucleic acids^39^. The decomposition of starch results in an increase in soluble sugar content.

### Effect of Cd stress on the activities of SOD, POD, CAT, and APX

Under heavy metals stress, excessive reactive oxygen species (ROS) are produced in cells. Production of ROS that exceeds the neutralizing capacity of cells leads to peroxidation, a type of oxidative stress that induces pathological processes and ultimately cell death^41^. The antioxidant system consists of two main parts; namely antioxidant enzymes and antioxidants. The main antioxidant enzymes include superoxide dismutase (SOD), peroxidase (POD), catalase (CAT), and other enzymes^42^. The antioxidants include mainly glutathione (GSH), free proline, ascorbic acid, malondialdehyde, etc.^43^. SOD, POD, CAT, and ascorbic acid peroxidase (APX) belong to heavy metals stress-induced enzymes reported by various studies^44–46^. SOD is the first line of defense against ROS, disproportionating O_2_^−^ into oxygen and H_2_O_2_. POD and CAT break down H_2_O_2_ to water and oxygen^16,47–49^, while APX is also an essential regulator of ROS scavenging through the ascorbate (ASA)-GSH cycle^40^. Generally, antioxidant enzymes exhibited an increase in response to lower levels, followed by a decline in response to higher metal stress^16,49–51^.

**Figure 3:**
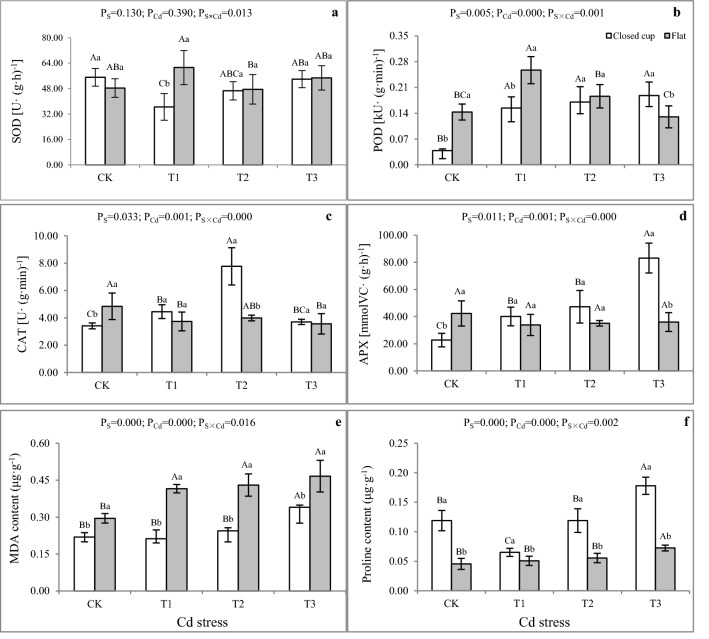
Effect of Cd on the activities of SOD, POD, CAT, and APX, and the content MDA and free proline in the fruiting bodies of *A. bisporus*. (**a**) SOD activity. (**b**) POD activity. (**c**) CAT activity. (**d**) APX activity. (**e**) MDA content. (**f**) Proline content. Different capital letters indicate significant differences among Cd stresses under the same sampling stage determined by LSD’s test with p < 0.05; different lower letters indicate significant differences within the sampling stage under the same Cd stress concentration determined by LSD’s test with p < 0.05. P_S_: effect of sampling time; P_Cd_: effect of Cd concentration; P_S×Cd_: Interaction between sampling time and Cd concentration. Error bar: SD.

In this study, as shown in Fig. 3, Cd-induced a strong antioxidant response in the fruiting body of *A. bisporus*, which caused the change in antioxidant enzyme activity. The SOD activities (Fig. 3a) showed an increasing trend with growth time in T1-T3 but decreased in the CK treatment. The SOD activity reached a maximum value of 61.23 U (g h)^−1^ in T1 in the flat stage (Fig. 3a), which was significantly higher than in CK, i.e., an increase of 26.69%. In the closed cup stage, the SOD activities increased with the increase of Cd content but were still lower than CK, which was similar to Cd stress in *A. brasiliensis* strain J1^38^. Here, SOD activity decreased with Cd stress content, indicating that SOD fails to be sufficient to detoxify O_2_^−^ to protect the plant from cellular damage which resulted in the generation of more O_2_^−^ under Cd stress^40^.

POD activities (Fig. 3b) under Cd stress were generally higher than those in CK; similar to changes in SOD activity in the closed cup stage. Nevertheless, POD activities decreased with increasing Cd stress in the flat stage. The maximum value of 0.26 kU (g min)^−1^ recorded in T1 was 79.32% higher than that in CK, and the difference was significant. CAT activities (Fig. 3c) showed a decreasing-rising trend with increasing Cd content. Its activities were higher than that in CK in the closed cup stage but lower than those in CK in the flat stage. Similarly, Cd stress reduced CAT enzyme activity in *stropharia rugosoannulata* mycelia^52^. With the prolongation of growth time, the CAT activities in CK increased, while in the Cd treatment group decreased. The maximum value of 7.76 U (g min)^−1^ appeared in T2 in the closed cup stage. In the closed cup stage, the SOD activity of the Cd treatments was lower than that in CK, while POD and CAT activities increased rapidly in the closed cup stage. The reason may be that the SOD has decomposed O_2_^−^ to H_2_O_2_, which leads to excessive accumulation of H_2_O_2_ in cells, thus increasing CAT and POD to decomposed H_2_O_2_, while decreasing SOD.

The changing trend of APX activities (Fig. 3d) with growth time was similar to that in CAT activity, in which APX of Cd treatments activities was higher than that in CK in the closed cup stage and lower than that in CK in the flat stage. With the increase of Cd content, the APX activity showed an upward trend in T1, T2, and T3, in which the records were 76.36%, 108.08%, and 266.05% higher than that in CK respectively in the closed cup stage. The maximum value of 83.14 mmolVC (g h)^−1^ appeared in T3 in the closed cup stage. The significant increase in APX activity indicates that the ASA-GSH cycle also plays a key role, which is a pathway for the conversion of H_2_O_2_ stimulated under Cd stress^53,54^. The accumulation of Cd in the fruiting body in the flat stage leads to cell damage with prolonged growth time, thereby inhibiting the synthesis of antioxidant enzymes. The Cd-treated CAT and APX activities were lower than that in CK, while POD activity decreased with increasing Cd, but was higher than that in CK compared to T1 and T2. Similar findings were previously revealed in *O. radicata*^49^, which found that POD and GR activity in the fruiting body of *O.radicata* decrease with increasing Pb stress, and higher than in CK compared to minimum Pb stress content of 250 mg kg^−1^. The final decrease in enzyme activities may be due to the hindrance of enzyme synthesis or changes in the accumulation of enzyme subunits caused by ROS access that lead to lipid peroxidation^55^.

### Effect of Cd stress on free proline and MDA

MDA content is a significant indicator of lipid peroxidation in plant cells as well as in the fruiting bodies of mushrooms^16,33^. MDA is the final decomposition product of membrane lipid peroxidation and its accumulation would cause severe damage to different membranes and cells^51^. In the study, the MDA contents (Fig. 3e) were 0.212–0.466 μg g^−1^. The flat stage was 34.83–95.93% higher than that in the closed cup stage with a significant difference (p < 0.05). In the flat stage, the MDA contents in T1, T2, and T3 were significantly higher than in CK, which were 40.80%, 43.83%, and 58.08% respectively. The MDA content increased with Cd stress and was higher than that in CK, except in T1 in the closed cup stage, which was in line with that in the *P. ostreatus* HAU-2^16^, which reported that the low content of Cd does not contribute to an increase of MDA content. The increase of MDA indicated that the membrane lipids of *A. bisporus* were oxidized by Cd stress, and the degree of lipid membrane peroxidation increased with the increase of stress time and content. Lipid membrane peroxidation could cause cell membrane damage and intracellular osmotic pressure loss of balance. The contents of the free proline (Fig. 3f) were 0.045–0.178 μg g^−1^, which increased with Cd stress. The closed cup stage was significantly higher than the flat stage. The free proline contents in T1 and T2 were lower than that in CK, but in T3 were 49.63% higher than that in CK with a significant difference (p < 0.05) in the closed cup stage. Whereas in the flat stage, the free proline contents under Cd stress were higher than that in CK.

The free proline maintains osmotic balance, stabilizes cell membranes to prevent electrolyte leakage, and reduces ROS levels^56^. In addition, free proline also acts as a heavy metals chelator to alleviate heavy metals stress^57^. Several fungi and plants have been reported to accumulate a high level of free proline under heavy metals stress, such as *Parthenium hysterophorus*^58^, and *Boletus edulis*^59^. The changing trend of free proline with Cd content was consistent with that in MDA, revealing that increased proline synthesis exhibited a protective effect against Cd toxicity by inhibiting lipid peroxidation. In the closed cup stage, the free proline content was higher than that in the flat stage. The results were similar to that in the *Cymbopogon flexuosus* Stap F., in which proline accumulation following short-term exposure might be higher than in long-term exposure under Pb, Hg, and Cd stress^60^.

### Effect of Cd stress on the contents of LMWOAs

**Figure 4:**
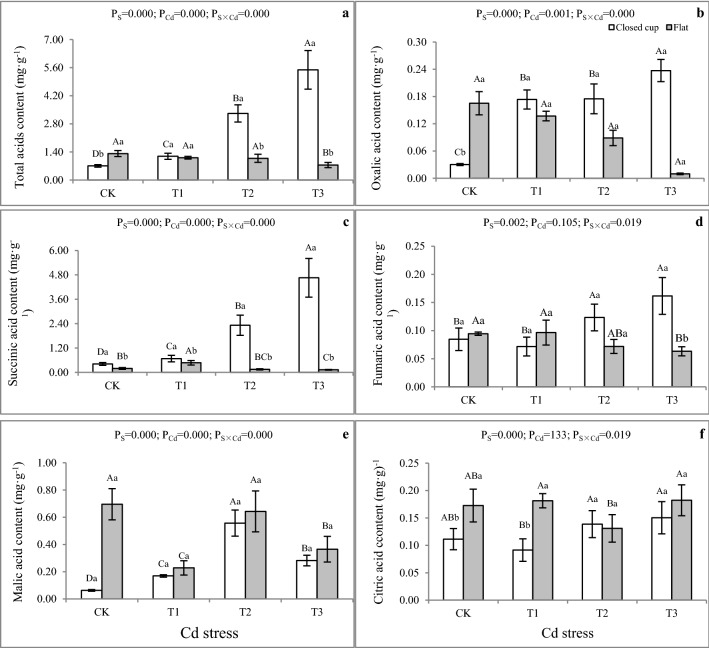
Effect of Cd stress on the amount of LOWOAs in fruiting bodies of *A. bisporus*. (**a**) Total organic acid content. (**b**) Oxalic acid content. (**c**) Succinic acid content. (**d**) Fumaric acid content. (**e**) Malic acid content. (**f**) Citric acid content. Different capital letters indicate significant differences among Cd stresses under the same sampling stage determined by LSD’s test with p < 0.05; different lower letters indicate significant differences within the sampling stage under the same Cd stress concentration determined by LSD’s test with p < 0.05. P_S_: effect of sampling time; P_Cd_: effect of Cd concentration; P_S×Cd_: Interaction between sampling time and Cd concentration. Error bar: SD.

The total contents of five LMWOAs (total acids) were 0.71–5.49 mg g^−1^ FW, with a significant difference under Cd stress. As shown in Fig. 4a, under Cd treatment, the total acid contents in the closed cup stage were higher than that in the flat stage, and the differences between T2 and T3 were significant (P < 0.05). With the increase of Cd stress, the total acid content increased in the closed cup stage and decreased in the flat stage. The changes in the trend of oxalic, succinic (except T1 treatment), and fumaric acid were similar to the total acids (Fig. 4c,d). Under the Cd stress, the contents of malic acid first increased and then decreased, while the contents in the flat stage were higher than that in the closed cup stage (Fig. 4e). The contents of citric acid in the flat stage were higher than that in the closed cup stage, and increased with the increase of Cd stress content, except in the T2 treatment (Fig. 4f). The order of the contents of five LMWOAs was basically: succinic acid > malic acid > citric acid > oxalic acid > fumaric acid (Fig. 4b–f). The succinic acid was the dominant organic acid in the fruiting body of *A. bisporus* at 13.79%-84.86%. The fumaric acid content was the lowest among LMWOAs at 2.84–11.97%. Overall, the contents of the total, oxalic, malic, formic (except T1), and succinic acids in Cd-exposed were higher than those in CK (P < 0.05) in the closed cup stage, whereas the levels of citric acid had a significant difference between CK and Cd treatments (P < 0.05). In the flat stage, the contents of LMWOAs in CK were higher than that in Cd treatment. However, those differences were not all significant. The LMWOAs also play an important role in tolerance to the stress of heavy metals in organisms. The organic acids secreted by roots can chelate complex heavy metals in the soil to improve the tolerance to heavy metals stress^61–65^. There are also a variety of organic acids in the cell that can transport, chelate, or complex heavy metals to reduce their toxic effect^66–68^. The increasing LMWOAs in the presence of Cd can affect chelation, thereby reducing the toxicity of the metal as well as enhancing its accumulation^69^*.*

In the present study, the total LMWOAs produced by *A. bisporus* in the closed cup stage under the Cd stress was 1.66–7.73 times higher than that in CK, suggesting their crucial role in reducing the damaging effect of Cd and improving the accumulation of the metal in *A. bisporus.* The results were in line with that of the *Agrocybe aegerita*^69^. Their study found that *A. aegerita* produced 1.49–2.08 times and 1.35–2.03 times LMWOAs (total acids) in pileus and stipe respectively in Cd-supplied soil compared to CK. When Cd enters the cell, LMWOAs can compartmentalize it into a vacuole based on metal–ligand complexation^70^. Vacuole contains many proteins, sugars, and organic acids, which can be combined with heavy metals to reduce their effectiveness^71,72^. The organic acids synthesized and secreted under heavy metals stress are related to the heavy metals type and species. The oxalic acid synthesis increased in Mn-exposed *Phytolacca americana* L.^29^. The secretion of oxalic, citric, and malic acids was enhanced with the treatment of Cd in *O. radicata*^30^. The sorghum enhanced malate exudation and maize increased mainly citrate under Cd stress^61^. In the present study, the content of total acids, oxalic, succinic, and fumaric acid increased in the closed cup stage and decreased in the flat stage under the Cd stress. The reason may be that: the short-term Cd exposure initiates the cell emergency mechanism and synthesizes LMWOAs to chelate with Cd to reduce its toxic effect; while the long-term Cd exposure inhibits the physiological and biochemical processes and induces cell damage, resulting in organic acid synthesis decline. The succinic, fumaric, malic, and citric acids are involved in the tricarboxylic acid cycle. Previously, an increase in phosphoenolpyruvate carboxylase (PEPC), citrate synthase, isocitrate dehydrogenase, and malate dehydrogenase activities also involved in the tricarboxylic acid cycle was found in Cd-exposed tomato plants, whereas fumarase activity showed a decline; thus indicating the tomato plant’s ability to accumulate Cd in roots is associated with the increased activity of the PEPC-malate dehydrogenase-citric acid synthetase (PEPC-MDH-CS) metabolic pathway involved in citric acid synthesis in roots^73^. Cd stress resulted in up to 400% higher expression of genes encoding three isozymes of plant-type PEPCs and up to 200% higher expression of genes encoding two isozymes of PEPC kinase in roots of Arabidopsis^74^. The content of oxalic acid, succinic acid, and fumaric acid was higher in the closed cup stage, while the content of malic acid and citric acid was higher in the flat stage, which indicated that the type of LMWOAs that played an important role in different stages of Cd stress would change, and the metabolism of organic acids was one of the mechanisms fungi use to tolerate heavy metals stress.

## Conclusions

The accumulation and detoxification of Cd, as well as a stress response in the fruiting body of *A. bisporus*, were analyzed in this study for the first time. *A. bisporus* can tolerate high content of Cd up to 414.28 ± 0.12 mg kg^−1^ and can accumulate Cd. The protein and sugar metabolism, various enzymatic and nonenzymatic antioxidants, and LMWOAs of *A. bisporus* played an important role in detoxification and survived under Cd stress. Generally, Cd stress caused oxidative damage in *A. bisporus*, which was confirmed by enhanced MDA. The *A. bisporus* resorts to modulating its metabolism to avoid the destructive effects of oxidative stress, including the increase of soluble protein, free proline, antioxidant enzyme activities, and LMOWOAs. Our results indicated that the physiological function of *A. bisporus* adjusted to Cd stress, showing its potential ability to serve as a hyper hyperaccumulator to remediate Cd-contaminated soils for the short lifetime of bioaccumulation.

## Supplementary Information

Supplementary Information.

## Data Availability

All data generated or analyzed during this study are included in this published article [and its supplementary information files].
